# Effects of carbon dioxide hydrate cryotherapy on knee flexor muscle function recovery trajectory after muscle damage: a randomized controlled trial

**DOI:** 10.3389/fphys.2026.1924770

**Published:** 2026-09-22

**Authors:** Rina Watabe, Daigo Shiozaki, Kohei Ono, Kosuke Hirata, Ryota Akagi

**Affiliations:** 1 Graduate School of Engineering and Science, Shibaura Institute of Technology, Saitama, Japan; 2 Department of Psychology, Columbia University, New York, NY, United States; 3 Institute of Health and Sport Sciences, University of Tsukuba, Tsukuba, Japan; 4 Sports Engineering Course, Bioscience and Engineering Program, College of Systems Engineering and Science, Shibaura Institute of Technology, Saitama, Japan

**Keywords:** eccentric exercise, hamstring muscles, maximal voluntary isometric contraction torque, muscle stiffness, pressure-induced muscle soreness

## Abstract

**Introduction:**

This study investigated the effects of carbon dioxide hydrate (CDH) cryotherapy on the recovery trajectories of muscle function and physiological markers following exercise-induced muscle damage (EIMD) of the knee flexors.

**Methods:**

In a randomized parallel-group design, 24 young men were assigned to the CDH, conventional icing (ICE), or non-intervention control (CON) group (n = 8 per group). To induce muscle damage, participants performed 10 sets of eight maximal eccentric contractions of the knee flexors in a prone position (knee joint range: 75°–15° of flexion at 60°/s; 0° = the fully extended anatomical position). Maximal voluntary isometric contraction (MVIC) torque of the knee flexors was measured at 30° of knee flexion at baseline (PRE), immediately post-exercise (POST), and at 4.5, 24, 48, 72, and 168 h post-exercise. Secondary outcomes, including thigh circumference, hip joint range of motion (ROM), and hamstring muscle stiffness and pressure-induced soreness were assessed at all time points except POST. A 20-min intervention was administered after each assessment from POST to 72 h.

**Results:**

Linear mixed-effects models, adjusted for PRE values, revealed no significant group × time interactions for changes from PRE in any outcome across post-exercise time points (POST to 168 h for MVIC torque; 4.5 to 168 h for secondary outcomes; *p* = 0.177–0.914). A significant main effect of group was observed only for hip joint ROM (*p* = 0.039). *Post-hoc* comparisons revealed a significantly greater overall reduction from PRE in the CDH group than in the CON group (difference: 10.3°, 95% CI: 3.0° to 17.6°, *p* = 0.017), with no significant differences between the other group pairs (both *p* = 0.131). Given the multi-outcome testing, this isolated effect should be interpreted as exploratory.

**Conclusion:**

Under the present experimental conditions, CDH cryotherapy did not accelerate the recovery of muscle function or attenuate secondary markers following EIMD relative to conventional icing or no intervention.

## 1 Introduction

Unaccustomed eccentric exercise induces muscle damage, which is characterized by a prolonged reduction in muscle strength, localized swelling, delayed-onset muscle soreness, and increased muscle stiffness (Clarkson et al., 1992; Ema et al., 2021). These physiological alterations associated with exercise-induced muscle damage (EIMD) substantially impair athletic performance (Horner et al., 2024) and may increase the risk of secondary injury during the recovery period (Garrett, 1996). Therefore, identifying effective recovery strategies is a critical challenge in applied sports science and exercise physiology.

Among the various recovery modalities, cryotherapy, or icing, has long been a foundational component of acute muscle damage management, traditionally incorporated into the Rest, Ice, Compression, and Elevation principle (Knight et al., 2000). The primary benefits of cryotherapy include reductions in acute inflammation and pain through decreases in tissue metabolism and nerve conduction velocity (Algafly and George, 2007). Indeed, cold-water immersion and systemic cryostimulation have been shown to reduce post-exercise muscle pain sensitivity, attenuate systemic markers of inflammation and muscle damage, and limit increases in muscle stiffness (Klich et al., 2018; Wolska et al., 2023). However, a fundamental limitation of conventional local cooling is its pronounced vasoconstrictive effect, which markedly reduces local blood flow (Mawhinney et al., 2013). Although cryotherapy is widely used in clinical practice, empirical evidence supporting its beneficial effects on human tissue regeneration remains scarce (Racinais et al., 2024), and animal studies have failed to demonstrate reductions in secondary injury following icing (Kawashima et al., 2021; Takagi et al., 2011). Rather, post-injury cooling may inadvertently impede the recovery process by delaying the necessary inflammatory response and subsequent cascade of muscle regeneration, potentially because of compromised microcirculation (Kawashima et al., 2021; Vieira Ramos et al., 2016). Thus, a major research gap remains regarding whether a cooling modality can provide anti-inflammatory and analgesic benefits without compromising local blood flow and functional muscle recovery.

Recently, carbon dioxide hydrate (CDH) cryotherapy has been proposed as a novel method for accelerating recovery from muscle fatigue (Hirata et al., 2020a, 2024). CDH is a gas hydrate clathrate (Smith et al., 2002) that combines thermal cooling with transcutaneous carbon dioxide-mediated vasodilation (Sakai et al., 2011). Consequently, CDH provides a cooling effect while substantially attenuating the reduction in local blood flow typically observed with conventional icing (Hirata et al., 2024). Although CDH therapy may retain the traditional benefits of cooling without compromising local tissue regeneration, its efficacy in facilitating recovery from EIMD remains unexplored.

Therefore, this study aimed to investigate the effects of CDH cryotherapy on the recovery trajectories of muscle function and symptoms of muscle damage following EIMD of the knee flexors, a muscle group subjected to substantial eccentric loading and highly susceptible to strain injuries during high-speed activities such as sprinting and kicking (Gronwald et al., 2022; Opar et al., 2012). We hypothesized that CDH cryotherapy would accelerate the recovery of muscle function, the primary outcome, compared with conventional icing and a non-intervention control. For the secondary outcomes, we expected that CDH application would also attenuate other associated symptoms of EIMD.

## 2 Materials and methods

### 2.1 Participants

Twenty-four young men were recruited and randomly assigned to one of three groups (n = 8 per group) in a 1:1:1 ratio: CDH cryotherapy (CDH group), conventional icing (ICE group), or non-intervention control (CON group). The randomization sequence was generated before study commencement by an investigator using Microsoft Excel 2021 (Microsoft, Redmond, WA, USA). To eliminate potential expectation bias or differential effort during the baseline (PRE) and acute exercise-induced damage phases, group allocations were strictly concealed from all participants throughout the PRE measurement, the muscle-damaging repetitive eccentric exercise protocol, and the immediate post-exercise (POST) assessment. Participants were informed of their allocated intervention only after completion of the POST measurements, immediately before the first intervention session. Because of the distinct physical characteristics of the thermal interventions, participant blinding during the subsequent intervention period was not feasible.

The primary outcome of this study was muscle function, as assessed by knee flexion torque during maximal voluntary isometric contraction (MVIC). MVIC torque measurements were conducted at seven time points: PRE, POST, and 4.5, 24, 48, 72, and 168 h post-exercise (4.5H, 24H, 48H, 72H, and 168H). The required sample size was determined through an *a priori* power analysis using G*Power (version 3.1.9.4; Kiel University, Kiel, Germany) based on a two-way repeated-measures analysis of variance (ANOVA) (within-between interaction) for the six post-exercise measurements. The input parameters were as follows: Effect size f = 0.25 (medium; Cohen, 1988); α err prob = 0.05; Power (1 – β err prob) = 0.80; Number of groups = 3; Number of measurements = 6; Corr among rep measurements = 0.50; and Nonsphericity correction e = 1. The analysis revealed that a sample size of 24 participants was required. Although linear mixed-effects models (LMMs) were used as the primary statistical method, an ANOVA-based power analysis was performed because G*Power does not explicitly support LMMs, and the ANOVA design provides a standard proxy for longitudinal studies. The demographic characteristics (mean ± standard deviation [SD]) of the participants in the different groups were as follows: CDH group (age: 22 ± 2 years, height: 169.7 ± 7.1 cm, body mass: 59.0 ± 9.0 kg), ICE group (age: 23 ± 1 years, height: 169.7 ± 5.5 cm, body mass: 60.0 ± 6.5 kg), and CON group (age: 23 ± 1 years, height: 170.0 ± 6.3 cm, body mass: 57.8 ± 7.2 kg). The exclusion criteria were a history of or current knee injury and participation in any lower-body resistance training within the previous 6 months. This study was approved by the Ethics Committee of Shibaura Institute of Technology (No. 23-037) and conducted in accordance with the guidelines outlined in the Declaration of Helsinki. Although the study was originally designed as a laboratory-based physiological experiment rather than a clinical trial and was not registered prospectively, it has now been retrospectively registered with the University Hospital Medical Information Network (UMIN) Clinical Trials Registry (UMIN000062804). The participants were informed of the study’s purpose and potential risks and provided written informed consent before participation.

### 2.2 Experimental procedures

All measurements were performed on the right leg of each participant, regardless of leg dominance, as the magnitude of muscle damage is not affected by leg dominance (Hody et al., 2013). MVIC torque was determined at all time points (PRE, POST, 4.5H, 24H, 48H, 72H, and 168H) using a dynamometer (CON-TREX MJ; PHYSIOMED, Schnaittach, Germany) connected to a 16-bit analog-to-digital converter (PowerLab16/35; ADInstruments, Bella Vista, Australia). Joint torque and angle data were recorded at a sampling frequency of 2 kHz using LabChart software (version 8.1.24; ADInstruments, Bella Vista, Australia). The remaining variables, including thigh circumference; stiffness and pressure-induced soreness of the biceps femoris long head (BF), semitendinosus (ST), and semimembranosus (SM) muscles; and hip joint range of motion (ROM), were designated as secondary outcomes and were measured at the same time points, except at POST. The 20-min intervention was then administered 5 min after completion of the measurements at five time points (POST, 4.5H, 24H, 48H, and 72H). The experimental room temperature was maintained at 23 ± 1 °C using an air-conditioning system throughout the measurement period. To minimize the influence of diurnal variation, the testing times for PRE and subsequent daily follow-up visits (24H, 48H, 72H, and 168H) were standardized to within ±1 h for each participant. An overview of the experimental timeline, including the timing of the measurements and interventions, is presented in **Figure 1**.

**Figure 1:**
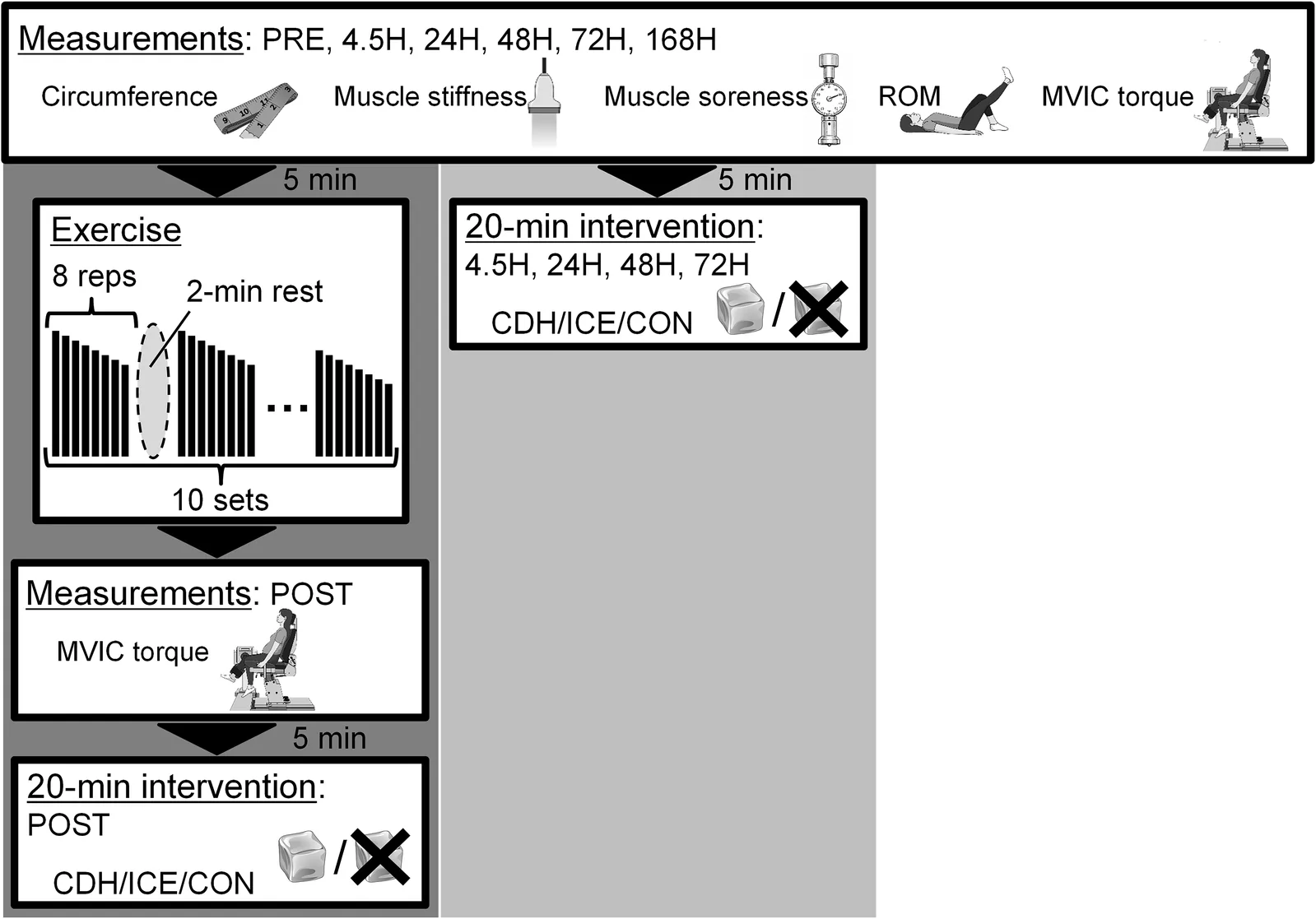
Timeline of the experimental protocol, including the timing of measurements and interventions. Seven time points were assessed: before exercise (PRE), immediately after the muscle-damaging repetitive eccentric exercise (POST), and at 4.5, 24, 48, 72, and 168 h post-exercise (4.5H, 24H, 48H, 72H, and 168H). Abbreviations are defined as follows: ROM, range of motion; MVIC, maximal voluntary isometric contraction; CDH, carbon dioxide hydrate cryotherapy; ICE, conventional icing; and CON, non-intervention control.

### 2.3 Circumference, muscle stiffness and pressure-induced soreness, and joint ROM

At PRE, while the participants stood upright, their thigh lengths were measured from the greater trochanter to the popliteal crease using a steel tape measure. Measurements were recorded to the nearest 0.5 cm. A mark was made at 50% of this length, measured from the greater trochanter. Subsequently, thigh circumference was measured at this mark using a cloth tape measure to the nearest 0.1 cm as an indicator of thigh edema (Chalchat et al., 2022).

The participants were positioned in a prone position on a bed with their ankles extending beyond the edge in a neutral position. In the mediolateral direction, the centers of the BF, ST, and SM muscles were identified using an ultrasound system (ACUSON Sequoia; Siemens Medical Solutions, Issaquah, WA, USA) at 50% of the thigh length and marked for the assessment of muscle stiffness and pressure-induced soreness.

Because increased muscle stiffness is a recognized indicator of muscle damage (Ema et al., 2021; Lacourpaille et al., 2014), the resting shear moduli of the BF, ST, and SM were then measured at 50% of the thigh length using longitudinal ultrasound shear wave elastography. Visualization of the muscle and tendon was optimized using the MSK Deep preset on the ultrasound system. The imaging gain was set to low (−20 dB), and the dynamic range was set to 65 dB to ensure optimal image quality. The mechanical index for the shear wave elasticity measurements was maintained at 0.80, and the speed of sound was set to 1,540 m/s.

A single examiner gently applied a 5cm linear-array transducer (15L4 Transducer; 3.5–15.1 MHz) with water-soluble gel to each muscle and acquired elastography images on a blue-to-red scale that reflected shear-wave propagation speed. Image quality was assessed on a separate green-to-orange color-coded scale, and images with ≤ 20% yellow-to-orange pixels within the region of interest (ROI) were considered acceptable. Measurements were repeated until three elastographic images of sufficient quality were obtained for each muscle in randomized order. All acquired images obtained were transferred to a personal computer in DICOM format.

Elastographic images were analyzed as previously described (Akagi et al., 2022, 2024). The ROI on each color map was made as large as possible while excluding nontarget tissues (e.g., subcutaneous adipose tissue, aponeuroses, and nontarget muscles) using ImageJ 1.52a (National Institutes of Health, Bethesda, MD, USA). The average shear-wave propagation speed within the ROI was computed for each image using analysis software written in MATLAB (version R2018a; MathWorks, Natick, MA, USA) (Hirata et al., 2020b). The coefficient of variation (CV) and intraclass correlation coefficient (ICC) for the mean of three measurements (ICC(1,3)) were calculated for shear-wave propagation speed to assess the examiner’s measurement precision and intrasession reliability. At PRE, the CV and ICC(1,3) for the three measurement across the 24 participants were 4.3% ± 3.2% and 0.964 (*p* < 0.001) for the BF, 2.9% ± 2.1% and 0.949 (*p* < 0.001) for the ST, and 3.2% ± 2.5% and 0.980 (*p* < 0.001) for the SM, respectively. The square of the average shear wave propagation speed across the three images was multiplied by the assumed muscle density of 1,084 kg/m³ (Ward and Lieber, 2005) to determine the muscle shear modulus (Nordez and Hug, 2010).

Because delayed-onset muscle soreness is a reliable indicator of muscle damage (Cheung et al., 2003), the extent of pressure-induced muscle soreness in the BF, ST, and SM was quantified at 50% of the thigh length using a visual analog scale (VAS) (Doguet et al., 2016; Ema et al., 2021), with one end labeled “no pain” (0 cm) and the other labeled “unbearable pain” (10 cm). The examiner applied a force of 80 N to the target muscle using a portable muscle hardness meter (NEUTONE TDM-N1; TRY-ALL Corp., Chiba, Japan) fitted with a half-cut rubber ball (diameter, 2.4 cm), and the participants rated their perceived pressure-induced soreness.

Because muscle damage reduces ROM (Clarkson et al., 1992), hip flexion ROM was measured using a digital angle gauge (AG01; HUEPAR Technology Co., Ltd., Shenzhen, China) attached to the ankle. The participants lay supine on a bed with the pelvis and left knee secured with straps. The examiner gently elevated the straight right leg until the participants reported reaching their limit of motion due to tension or pain. The resulting hip flexion angle was recorded, and the mean of three trials was used. The intrasession reliability of hip flexion ROM at PRE across the 24 participants was high, with a CV of 3.2% ± 1.7% and an ICC(1,3) of 0.992 (*p* < 0.001).

The marks placed at PRE were retained for use at the subsequent time points. Measurements of circumference, muscle stiffness and pressure-induced soreness, and ROM were repeated at all time points, except POST.

### 2.4 MVIC torque

The participants were positioned in a prone position on the dynamometer bed, with their hips and knees flexed to 30° (0° was defined as the anatomical position). One or more sheets of extruded polystyrene foam, each 3 cm thick, were placed between the bed and the participants to align the axis of rotation of the knee joint with that of the dynamometer and ensure proper body positioning. The participants’ trunks were secured to the bed with straps.

At all time points except POST, following a warm-up consisting of 2–3 submaximal contractions, peak torque during a 3-s MVIC of the knee flexors was determined twice with a 1-min rest interval. If the difference between the two peak torque values was > 10% of the higher torque, additional contractions were performed until the difference between the highest and second-highest values was < 10% of the highest torque. Strong verbal encouragement was provided during each MVIC. The highest peak torque was defined as the MVIC torque. During offline analysis, the torque data were low-pass filtered at 500 Hz using a standard Butterworth infinite impulse response filter implemented in LabChart software (ADInstruments).

At POST, MVIC torque was measured once, immediately following the eccentric exercise, under the same conditions as at all other time points to ensure a prompt and consistent transition to the intervention phase.

### 2.5 Eccentric exercise

After a 5-min rest following the PRE MVIC torque measurements, the participants performed 10 sets of eight repetitions of maximal eccentric contractions of the knee flexors in the same posture as that used for the MVIC torque measurements (knee joint angle range: 75°–15° of flexion at 60°/s; passive return at 12°/s; 5-s rest between repetitions; 2-min rest between sets). The participants received verbal encouragement to resist the movement as much as possible.

### 2.6 Intervention

In the CDH and ICE groups, the knee flexors were cooled for 20 min (from 5 to 25 min after the end of the measurements) at POST, 4.5H, 24H, 48H, and 72H, using 200 g of CDH or conventional ice, respectively. Both CDH and conventional ice packs were stored in a temperature-controlled freezer at −20 °C until immediately before application, providing a mass-matched (200 g, 20 min) protocol designed for practical use in sports and clinical settings. During the intervention, the participants remained relaxed in the same prone position as that used for the MVIC measurements. A support stand was provided for the non-intervention (left) leg to ensure complete relaxation. CDH or conventional ice was placed directly on the knee flexors, centered at 50% of the thigh length, to cover the area where the hamstring muscles were most prominent. A thermally insulated frame made of extruded polystyrene foam (20 cm long × 10 cm wide) was used to contain the CDH or ice. The frame initially had a thickness of 6 cm and was carefully shaped to conform to the thigh contour, resulting in a minimum depth of 3 cm. After the cooling material was placed within the frame, a lid made of the same foam was applied to seal the area and maximize thermal insulation. This setup prevented the loss of cooling capacity during the 20-min intervention period. Conventional ice was prepared from pure water without carbon dioxide, whereas CDH contained approximately 10% carbon dioxide by weight (NIPPON EKITAN Corporation, Tokyo, Japan). In the CON group, the participants assumed the same posture as that used in the other two groups and maintained it for 20 min without muscle icing.

### 2.7 Statistical analyses

All statistical analyses were performed using JASP (version 0.98.1; JASP Team, Amsterdam, The Netherlands) and Excel (2021; Microsoft, Redmond, WA, USA). Data are presented as the mean ± SD. Statistical significance was set at *p* < 0.05.

All variables were analyzed as change scores (absolute changes from PRE). LMMs were conducted with fixed factors of group (CDH, ICE, and CON; between-subjects), time (within-subjects), and their interaction (group × time). Type III tests were used to evaluate for fixed effects, and degrees of freedom were approximated using Satterthwaite’s method. To avoid model misspecification due to zero variance at baseline, PRE was excluded from the repeated time factor, and the absolute baseline (PRE) value of each outcome was included as a covariate in all models. The time factor included POST, 4.5H, 24H, 48H, 72H, and 168H for MVIC torque, and 4.5H, 24H, 48H, 72H, and 168H for all other variables. To account for within-subject correlation across repeated measurements, participant identity was specified as a random intercept in all models. This specification assumed compound symmetry for the covariance structure across post-exercise time points. When a significant main effect of group or time was observed without a significant interaction, *post hoc* comparisons were performed to identify specific group or time differences. To control inflation of the Type I error rate across all *post-hoc* pairwise comparisons, *p* values were adjusted using the Benjamini–Hochberg false discovery rate correction. Regardless of the significance of the interaction, changes from PRE at each post-exercise time point were considered statistically significant within each group if the 95% confidence interval (CI) for the estimated change derived from the LMM did not include zero.

## 3 Results

### 3.1 MVIC torque

At PRE, MVIC torque was 117.6 ± 19.9 Nm in the CDH group, 113.6 ± 20.3 Nm in the ICE group, and 101.5 ± 16.4 Nm in the CON group. At POST, MVIC torque decreased from PRE by 37% ± 9% (PRE-adjusted estimated marginal mean [EMM]: −40.1 Nm; 95% CI: −51.2 to −29.0 Nm) in the CDH group, 28% ± 14% (EMM: −30.7 Nm; 95% CI: −41.7 to −19.7 Nm) in the ICE group, and 22% ± 16% (EMM: −27.8 Nm; 95% CI: −39.1 to −16.6 Nm) in the CON group, with an overall mean reduction of 29% across all participants. Specifically, 23 of 24 participants exhibited immediate torque decrements following the eccentric protocol, with only one participant in the CON group failing to show a torque reduction.

The LMM analysis revealed no significant group × time interaction (*F*[10, 105] = 1.254, *p* = 0.266) or main effect of group (*F*[2, 20] = 0.009, *p* = 0.991) for changes in MVIC torque from PRE across the post-exercise period (POST to 168H) (**Figure 2**). A significant main effect of time was observed (*F*[5, 105] = 8.793, *p* < 0.001). *Post hoc* pairwise comparisons for the main effect of time revealed no significant differences among POST, 4.5H, and 168H or among 24H, 48H, and 72H (*p* = 0.157–0.813), whereas all other pairwise comparisons between time points were statistically significant (*p* ≤ 0.029). Furthermore, evaluation of the 95% CIs indicated that MVIC torque remained significantly reduced from PRE at all post-exercise time points (POST to 168H) across all groups. The baseline-adjusted between-group differences are provided in **Supplementary Table S1**.

**Figure 2:**
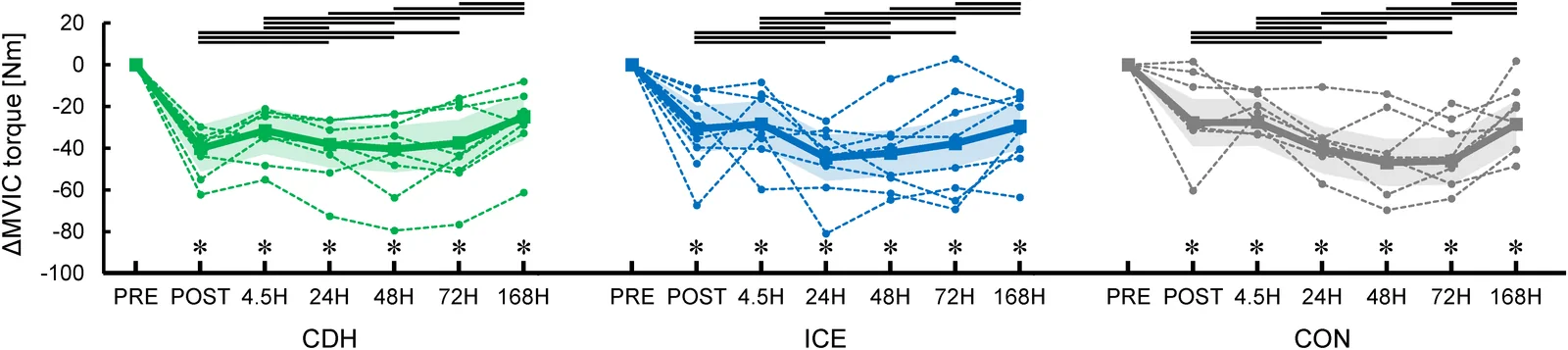
Time course of changes in knee flexion torque during maximal voluntary isometric contraction (MVIC) from baseline (PRE) in the carbon dioxide hydrate cryotherapy (CDH; green), conventional icing (ICE; blue), and non-intervention control (CON; gray) groups (n = 8 per group). Data are presented as individual participant values (dashed lines with circles) and estimated marginal means (solid lines with squares). Shaded bands represent the corresponding 95% confidence intervals (CIs) derived from the linear mixed-effects model adjusted for PRE MVIC torque. Measurements were performed at seven time points: PRE, immediately after the muscle-damaging repetitive eccentric exercise (POST), and at 4.5, 24, 48, 72, and 168 h post-exercise (4.5H, 24H, 48H, 72H, and 168H). In the absence of a significant group × time interaction, horizontal solid black lines at the top of the graph indicate significant pairwise differences between post-exercise time points for the main effect of time (*p* < 0.05). Asterisk (*) indicates significant changes from PRE within each group based on the 95% CIs (i.e., when the 95% CI does not include zero). Note that a difference in asterisk placement between panels does not indicate a statistically significant between-group difference.

### 3.2 Circumference

At PRE, thigh circumference was 49.2 ± 2.2 cm in the CDH group, 48.8 ± 3.9 cm in the ICE group, and 48.2 ± 3.7 cm in the CON group. **Figure 3** illustrates the time course of changes in thigh circumference from PRE across all groups.

**Figure 3:**
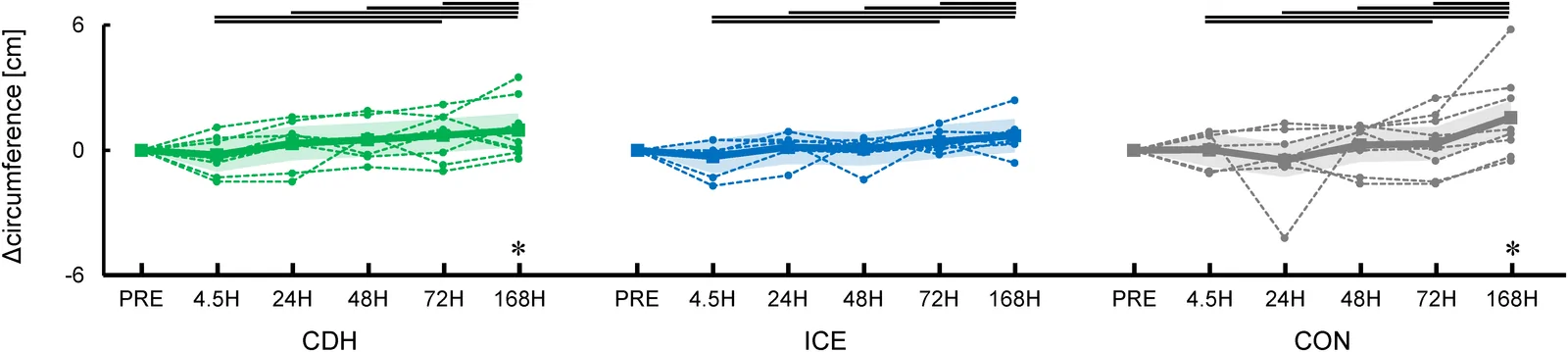
Time course of changes in thigh circumference from baseline (PRE) in the carbon dioxide hydrate cryotherapy (CDH; green), conventional icing (ICE; blue), and non-intervention control (CON; gray) groups (n = 8 per group). Data are presented as individual participant values (dashed lines with circles) and estimated marginal means (solid lines with squares). Shaded bands represent the corresponding 95% confidence intervals (CIs) derived from the linear mixed-effects model adjusted for PRE thigh circumference. Measurements were performed at six time points: PRE and at 4.5, 24, 48, 72, and 168 h post-exercise (4.5H, 24H, 48H, 72H, and 168H). In the absence of a significant group × time interaction, horizontal solid black lines at the top of the graph indicate significant pairwise differences between post-exercise time points for the main effect of time (*p* < 0.05). Asterisk (*) indicates significant changes from PRE within each group based on the 95% CIs (i.e., when the 95% CI does not include zero). Note that a difference in asterisk placement between panels does not indicate a statistically significant between-group difference.

The LMM analysis revealed no significant group × time interaction (*F*[8, 84] = 1.304, *p* = 0.253) or main effect of group (*F*[2, 20] = 0.152, *p* = 0.860) for changes in thigh circumference. A significant main effect of time was observed (*F*[4, 84] = 8.837, *p* < 0.001). *Post-hoc* pairwise comparisons for the main effect of time revealed that changes in thigh circumference at 168H were significantly greater than those at all earlier time points (4.5H to 72H; *p* ≤ 0.019) and those at 72H were significantly greater than those at 4.5H (*p* = 0.013), with no other significant differences between time points (*p* = 0.062–0.469). Furthermore, evaluation of the 95% CIs indicated that thigh circumference was significantly increased from PRE only at 168H in the CDH and CON groups, with no other significant changes from PRE observed across time points. The baseline-adjusted between-group differences are provided in **Supplementary Table S2**.

### 3.3 Muscle stiffness

At PRE, the muscle shear moduli in the CDH, ICE, and CON groups were 3.83 ± 0.75 kPa, 4.12 ± 1.86 kPa, and 3.68 ± 1.03 kPa for the BF; 3.26 ± 0.36 kPa, 3.86 ± 0.87 kPa, and 3.16 ± 0.40 kPa for the ST; and 3.58 ± 0.88 kPa, 4.06 ± 1.63 kPa, and 2.97 ± 1.02 kPa for the SM, respectively. **Figure 4** shows the time course of changes in the shear moduli of the BF, ST, and SM from PRE across all groups.

**Figure 4:**
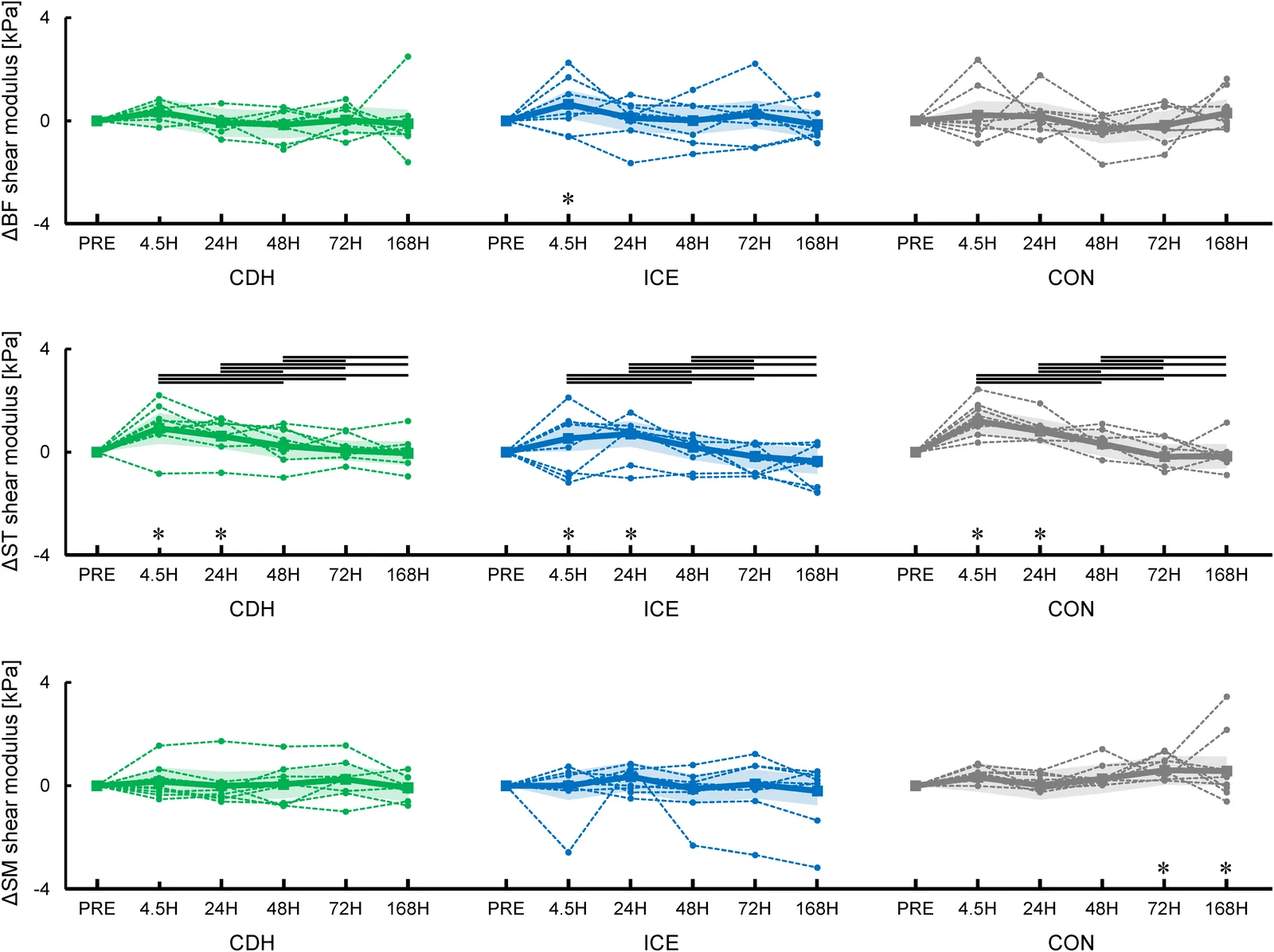
Time course of changes in the muscle shear moduli of the long head of the biceps femoris (BF), semitendinosus (ST), and semimembranosus (SM) from baseline (PRE) in the carbon dioxide hydrate cryotherapy (CDH; green), conventional icing (ICE; blue), and non-intervention control (CON; gray) groups (n = 8 per group). Data are presented as individual participant values (dashed lines with circles) and estimated marginal means (solid lines with squares). Shaded bands represent the corresponding 95% confidence intervals (CIs) derived from the linear mixed-effects models adjusted for PRE muscle shear modulus for each respective muscle. Measurements were performed at six time points: PRE and at 4.5, 24, 48, 72, and 168 h post-exercise (4.5H, 24H, 48H, 72H, and 168H). In the absence of a significant group × time interaction, horizontal solid black lines at the top of the graphs indicate significant pairwise differences between post-exercise time points for the main effect of time (*p* < 0.05). Asterisk (*) indicates significant changes from PRE within each group based on the 95% CIs (i.e., when the 95% CI does not include zero). Note that a difference in asterisk placement between panels does not indicate a statistically significant between-group difference.

No significant group × time interaction was observed for any of the three muscles (*F*[8, 84] = 0.669–1.129, *p* = 0.353–0.718). Similarly, no significant main effect of group was observed for any of the three muscles (*F*[2, 20] = 0.288–0.643, *p* = 0.536–0.753). A significant main effect of time was observed for the ST (*F*[4, 84] = 21.650, *p* < 0.001), whereas no significant main effect of time was observed for either the BF or SM (*F*[4, 84] = 0.611–2.097, *p* = 0.088–0.656). *Post hoc* pairwise comparisons for the main effect of time in the ST revealed that the change in shear modulus peaked at 4.5H and progressively decreased thereafter, with all pairwise comparisons between time points being significant (*p* ≤ 0.023), except those between 4.5H and 24H (*p* = 0.262) and between 72H and 168H (*p* = 0.518). Furthermore, evaluation of the 95% CIs indicated that the shear modulus of the ST was significantly increased from PRE at 4.5H and 24H across all groups. For the BF shear modulus, a significant increase from PRE was observed only at 4.5H in the ICE group, whereas significant increases in the SM shear modulus were observed only at 72H and 168H in the CON group. No other significant changes from PRE were identified for any muscle. The baseline-adjusted between-group differences are provided in **Supplementary Table S3**.

### 3.4 Pressure-induced muscle soreness

At PRE, VAS scores in the CDH, ICE, and CON groups were 3.6 ± 2.2 cm, 3.0 ± 1.9 cm, and 3.3 ± 1.2 cm for the BF; 3.8 ± 1.4 cm, 3.4 ± 2.1 cm, and 3.7 ± 1.3 cm for the ST; and 3.7 ± 1.5 cm, 2.1 ± 1.9 cm, and 4.1 ± 1.2 cm for the SM, respectively. **Figure 5** illustrates the time course of changes in VAS scores for the BF, ST, and SM from PRE across all groups.

**Figure 5:**
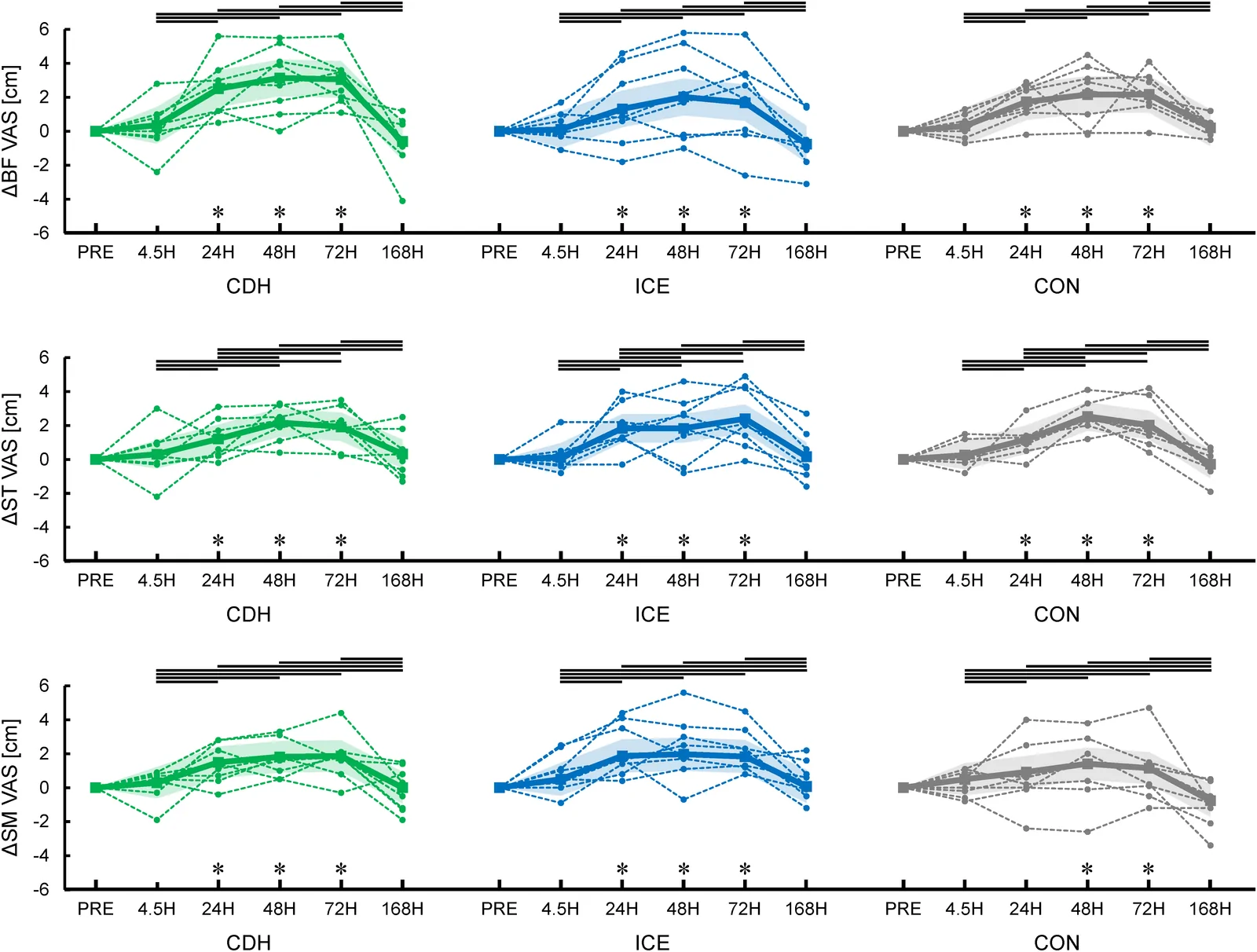
Time course of changes in pressure-induced muscle soreness assessed using a visual analog scale (VAS) in the long head of the biceps femoris (BF), semitendinosus (ST), and semimembranosus (SM) from baseline (PRE) in the carbon dioxide hydrate cryotherapy (CDH; green), conventional icing (ICE; blue), and non-intervention control (CON; gray) groups (n = 8 per group). Data are presented as individual participant values (dashed lines with circles) and estimated marginal means (solid lines with squares). Shaded bands represent the corresponding 95% confidence intervals (CIs) derived from the linear mixed-effects models adjusted for PRE VAS scores for each respective muscle. Assessments were performed at six time points: PRE and at 4.5, 24, 48, 72, and 168 h post-exercise (4.5H, 24H, 48H, 72H, and 168H). In the absence of a significant group × time interaction, horizontal solid black lines at the top of the graphs indicate significant pairwise differences between post-exercise time points for the main effect of time (*p* < 0.05). Asterisk (*) indicates significant changes from PRE within each group based on the 95% CIs (i.e., when the 95% CI does not include zero). Note that a difference in asterisk placement between panels does not indicate a statistically significant between-group difference.

The LMM analysis revealed no significant group × time interaction (*F*[8, 84] = 0.407–1.074, *p* = 0.389–0.914) or main effect of group (*F*[2, 20] = 0.052–0.926, *p* = 0.413–0.950) for changes in VAS scores for any of the three muscles. A significant main effect of time was observed for all three muscles (*F*[4, 84] = 17.040–31.500, *p* < 0.001). *Post hoc* pairwise comparisons revealed that for the BF, changes in VAS scores at 24H, 48H, and 72H were significantly greater than those at 4.5H and 168H (*p* < 0.001). For the ST, changes in VAS scores at 48H and 72H were significantly greater than those at 4.5H, 24H, and 168H, and changes at 24H were significantly greater than at 4.5H and 168H (*p* ≤ 0.018). For the SM, changes in VAS scores at 24H, 48H, and 72H were significantly greater than those at 4.5H, whereas changes at 168H were significantly smaller than those at all earlier time points (4.5H to 72H; *p* ≤ 0.036). Evaluation of the 95% CIs indicated that VAS scores for both the BF and ST were significantly increased from PRE at 24H, 48H, and 72H across all groups. For the SM, significant increases from PRE were observed at 24H, 48H, and 72H in the CDH and ICE groups and at 48H and 72H in the CON group, with no other significant changes from PRE identified for any muscle. The baseline-adjusted between-group differences are provided in **Supplementary Table S4**.

### 3.5 Joint ROM

At PRE, hip flexion ROM in the CDH, ICE, and CON groups was 52.0 ± 14.2°, 62.1 ± 11.4°, and 67.8 ± 13.1°, respectively. **Figure 6** presents the time course of changes in hip flexion ROM from PRE across all groups.

**Figure 6:**
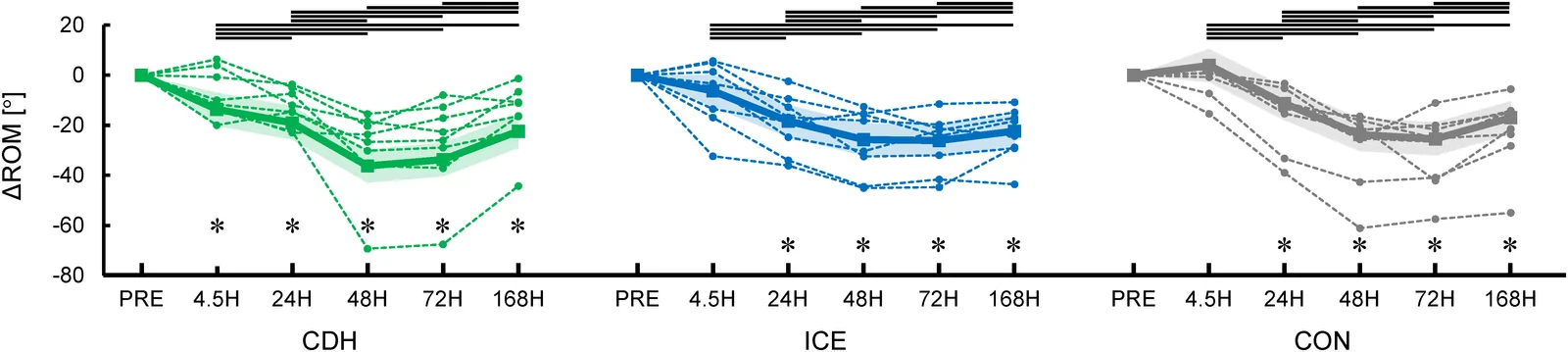
Time course of changes in hip flexion range of motion (ROM) from baseline (PRE) in the carbon dioxide hydrate cryotherapy (CDH; green), conventional icing (ICE; blue), and non-intervention control (CON; gray) groups (n = 8 per group). Data are presented as individual participant values (dashed lines with circles) and estimated marginal means (solid lines with squares). Shaded bands represent the corresponding 95% confidence intervals (CIs) derived from the linear mixed-effects model adjusted for PRE hip flexion ROM. Measurements were performed at six time points: PRE and at 4.5, 24, 48, 72, and 168 h post-exercise (4.5H, 24H, 48H, 72H, and 168H). In the absence of a significant group × time interaction, horizontal solid black lines at the top of the graph indicate significant pairwise differences between post-exercise time points for the main effect of time (*p* < 0.05). Asterisk (*) indicates significant changes from PRE within each group based on the 95% CIs (i.e., when the 95% CI does not include zero). Note that a difference in asterisk placement between panels does not indicate a statistically significant between-group difference.

The LMM analysis revealed no significant group × time interaction (*F*[8, 84] = 1.479, *p* = 0.177), but significant main effects of group (*F*[2, 20] = 3.843, *p* = 0.039) and time (*F*[4, 84] = 42.180, *p* < 0.001) were observed. Pooled across post-exercise time points, the EMM reduction from PRE was −25.0° in the CDH group, −19.8° in the ICE group, and −14.7° in the CON group. *Post-hoc* comparisons for the main effect of group revealed that the reduction from PRE was significantly greater in the CDH group than in the CON group (difference: 10.3°, 95% CI: 3.0° to 17.6°, *p* = 0.017), whereas no significant differences were found between the other group pairs (both *p* = 0.131). The fitted coefficient for the PRE covariate was β = −0.724 (95% CI: −0.954 to −0.494, *p* < 0.001). Because this was the sole significant main effect of group among the nine outcomes tested and no adjustment was applied across outcome measures, this finding should be interpreted as exploratory. For the main effect of time, reductions from PRE were smallest at 4.5H, followed by those at 24H and 168H, and were greatest at 48H and 72H. Pairwise comparisons revealed significant differences between all time points (*p* ≤ 0.045), except between 48H and 72H (*p* = 0.949). Evaluation of the 95% CIs indicated that hip flexion ROM was significantly decreased from PRE at all post-exercise time points across all groups, except at 4.5H in the ICE and CON groups. The baseline-adjusted between-group differences are provided in **Supplementary Table S5**.

## 4 Discussion

In this study, LMM analyses revealed no significant group × time interactions for the primary outcome (i.e., MVIC torque) or any of the secondary outcomes. Furthermore, no significant main effect of group was observed for MVIC torque or any secondary outcome, with the sole exception of hip flexion ROM, for which the overall reduction from PRE was significantly greater in the CDH group than in the CON group. Therefore, CDH cryotherapy neither accelerated the recovery of muscle function nor attenuated the associated symptoms of EIMD. Consequently, these findings do not support the primary or secondary hypothesis under the present experimental conditions.

Characterizing the severity of the initial muscle damage is critical for contextualizing the subsequent recovery patterns. Across all three groups, an overall mean reduction of 29% from PRE in MVIC torque was observed at POST. This magnitude of initial MVIC torque loss is comparable to that reported in previous studies that used similar eccentric exercise models of the knee flexors (Brusco et al., 2022; Chen et al., 2023, 2025), indicating that the present protocol successfully induced a substantial degree of EIMD. However, although no significant group × time interaction or main effect of group was observed for changes in MVIC torque from PRE, the numerical reduction at POST was somewhat greater in the CDH group (37% ± 9% [EMM: −40.1 Nm]) than in the ICE (28% ± 14% [EMM: −30.7 Nm]) and CON (22% ± 16% [EMM: −27.8 Nm]) groups. This numerical discrepancy may partly reflect the substantial interindividual variability inherently associated with EIMD responses and recovery trajectories, as has been well documented even among individuals exhibiting similar immediate post-exercise MVIC losses (Shoji et al., 2021). Furthermore, to facilitate a rapid transition to the assigned intervention immediately following the eccentric exercise protocol, POST MVIC torque was evaluated using a single trial, whereas at all other time points (including PRE), the higher value from at least two trials was used. This methodological constraint, combined with interindividual variability in initial susceptibility to damage, could have amplified the initial reduction in certain participants. Additionally, given this high degree of interindividual variability, our *a priori* power calculation targeting a medium effect size of f = 0.25 (which determined the total requirement of 24 participants as detailed in Section 3.1) might have resulted in a sample size per group insufficient to fully account for such subtle initial imbalances. These factors leave open the question of whether a comparable degree of initial muscle damage was induced across all groups. Therefore, these potential subtle imbalances in initial damage severity and differences in the measurement protocol must be carefully considered when interpreting the subsequent recovery trajectories.

Regarding the primary outcome, MVIC torque has been established as one of the most reliable indirect markers of EIMD severity, directly reflecting the overall contractile function of the muscle (Chalchat et al., 2022). Furthermore, restoration of muscle function is considered the most critical outcome for evaluating the efficacy of recovery interventions because it directly relates to an individual’s readiness to resume physical performance (Peake et al., 2017). In the present study, no significant group × time interaction or main effect of group was observed for changes in MVIC torque from PRE across the post-exercise period (**Figure 2**). Furthermore, MVIC torque did not fully recover to PRE values even by 168H post-exercise across all groups, underscoring the prolonged duration of functional impairment caused by the EIMD protocol used in this study. These findings indicate that conventional icing did not impair the recovery of muscle function and that CDH cryotherapy did not accelerate functional recovery compared with conventional icing or the non-intervention control. Furthermore, evaluation of the baseline-adjusted between-group differences and their 95% CIs (**Supplementary Table S1**) indicates that the true differences between interventions are bounded within approximately ±16 Nm across time points. This precision establishes the plausible range of potential intervention effects compatible with our data, precluding the presence of large differences while confirming that any subtle true benefits must lie within these limits.

In the present study, the local cooling duration was set to 20 min. This protocol was selected based on our previous finding that a single 20-min session of CDH cryotherapy significantly facilitated the recovery of joint power following fatigue-inducing exercise while attenuating the excessive microvascular constriction typically induced by conventional icing (Hirata et al., 2024). Furthermore, a 20-min duration falls within the evidence-based recommendation of 20–30 min per session for acute musculoskeletal injury management (Kwiecien, 2023). From a practical standpoint, extending the application time beyond 20 min would also reduce its feasibility and likely decrease adherence in real-world sports settings. Therefore, a single 20-min cooling intervention represented a standardized and clinically realistic dose for testing the recovery hypotheses in the present experimental model. These results align with concerns that the efficacy of cryotherapy in promoting functional regeneration in humans remains equivocal, despite its status as a clinical standard (Racinais et al., 2024). Mechanistically, much of our understanding of cooling-induced muscular responses is derived from laboratory animal models, which have yielded contrasting findings depending on the cooling dose and injury severity. For instance, a single 20-min icing application administrated soon after a severe crush injury in rats was reported to delay initial inflammation, retard satellite cell activity and muscle fiber regeneration, and lead to excessive collagen deposition (Takagi et al., 2011), whereas frequent, repeated 30-min cooling applications stimulated regeneration in models with minimal myofiber necrosis (Kawashima et al., 2024). The lack of a clear facilitative effect in the ICE group is consistent with previous suggestions that vasoconstriction induced by conventional cooling may offset potential recovery benefits by restricting the delivery of regenerative cells and nutrients to the injury site (Kawashima et al., 2021; Vieira Ramos et al., 2016). In the context of the present human EIMD model, which induced a noninvasive, physiological level of muscle damage (mean MVIC reduction of ~29%), a single 20-min exposure was neither sufficiently intense nor excessively prolonged to induce the severe regenerative impairment observed in animal models of severe crush injury, which may explain why ICE did not negatively affect recovery compared with CON.

Similarly, although CDH provided sustained cooling effect, a single 20-min session was likely insufficient to overcome these physiological constraints in the present study. This discrepancy from our previous findings, in which a single 20-min CDH session successfully facilitated recovery after a fatigue-inducing protocol (Hirata et al., 2024), may be attributable to the greater degree of structural muscle damage induced by the EIMD protocol compared with that associated with neuromuscular fatigue. Furthermore, as demonstrated in animal models of mild muscle damage (Kawashima et al., 2024), accelerating tissue repair may require frequent, repeated cooling applications rather than a single session. Given that effective management of acute injuries often requires sustained or intermittent interventions (Auley, 2001; Kwiecien, 2023), a single cooling session may be inadequate to modulate the prolonged inflammatory cascade associated with EIMD. Therefore, repeated cooling applications (e.g., intermittent sessions within the acute phase), rather than a single session, might be necessary for CDH cryotherapy to exert a measurable facilitative effect on functional recovery following EIMD.

Regarding the secondary outcomes, hip flexion ROM was the only parameter that showed a significant main effect of group, with the CDH group exhibiting a significantly greater overall reduction from PRE than the CON group (difference: 10.3°, 95% CI: 3.0° to 17.6°). However, no significant group × time interaction was observed for hip flexion ROM, nor were there any significant pairwise differences between the CON and ICE groups or between the ICE and CDH groups. It is worth noting that the CDH group had a lower baseline ROM (52.0 ± 14.2°) than the CON group (67.8 ± 13.1°). When examining the unadjusted mean reductions, the three groups showed similar changes (−18.8° in the CDH group, −20.9° in the ICE group, and −19.9° in the CON group), and the adjusted group difference largely reflects the statistical covariate adjustment acting on this 15.8° baseline gap. Nevertheless, this magnitude of difference is unlikely to represent a clinically meaningful physiological impairment. For all other secondary outcomes, no significant group × time interactions or main effects of group were observed. These consistent null findings across all other measures indicate that 20-min sessions of local cooling (either CDH cryotherapy or conventional icing) did not alter the overall post-exercise trajectory of EIMD markers. Because this was the sole significant group effect among the nine outcomes tested and was not adjusted across outcome measures, this isolated finding should be interpreted as exploratory.

Regarding muscle stiffness and pressure-induced muscle soreness, although neither CDH cryotherapy nor conventional icing produced a significant group × time interaction or main effect of group, distinct muscle-dependent temporal patterns were observed among the three hamstring muscles (**Figures 4**, **5**). For muscle stiffness (as assessed by muscle shear modulus), the LMM analysis revealed a significant main effect of time exclusively for the ST, whereas no significant main effect of time was observed for either the BF or SM. In the ST, the shear modulus peaked at 4.5H and remained significantly increased from PRE through 24H post-exercise across all groups before progressively declining. Although minor isolated increases from PRE were observed in specific groups for the BF (at 4.5H in the ICE group) and SM (at 72H and 168H in the CON group), this selective stiffening of the ST aligns with previous observations of muscle-specific hamstring responses following eccentric exercise (Goreau et al., 2022; Voglar et al., 2022). Several biomechanical and structural factors likely account for this marked susceptibility of the ST. First, the ST plays a predominant role in load-bearing during knee flexion tasks regardless of contraction mode (Evangelidis et al., 2021) and is preferentially activated during eccentric knee flexion exercises such as the Nordic hamstring exercise compared with its synergists (Bourne et al., 2016, 2017). Second, the fusiform architecture of the ST results in greater muscle fiber strain compared with the pennate architecture of the SM, rendering the ST more vulnerable to mechanical microdamage and subsequent stiffening (Goreau et al., 2022). Third, because the ST has a smaller muscle volume than the SM (Akagi et al., 2020) but exhibits greater neural activation (Ono et al., 2010), it is subjected to a greater relative mechanical load during maximal eccentric contractions. Conversely, the absence of an overall main effect of time in the BF may be attributed to its lower neural recruitment as the knee approaches full extension during eccentric knee flexion (Higashihara et al., 2010).

Physiologically, the acute increase in ST shear modulus at 4.5H may primarily reflect disrupted calcium homeostasis and the formation of residual cross-bridges (Lacourpaille et al., 2014), whereas its persistence through 24H may be driven by delayed fluid accumulation and edema (Dourado et al., 2023). In contrast, the isolated later increase in SM shear modulus observed at 72H and 168H may reflect delayed secondary inflammatory processes and peak fluid accumulation (Dourado et al., 2023). Alternatively, this late response could represent a biomechanical compensatory strategy during daily functional movements as the markedly stiffened ST begins to recover. Unlike muscle stiffness, pressure-induced muscle soreness showed a significant main effect of time across all three hamstring muscles, with VAS scores substantially increased during the 24H–72H post-exercise period. This distinct temporal dissociation between ST stiffening (peaking at 4.5H and remaining increased through 24H) and pressure-induced muscle soreness (substantially increased at 24H–72H across all three muscles) indicates that early mechanical stiffening and delayed inflammatory nociceptive sensitization may represent distinct physiological processes following EIMD. Taken together, these findings indicate that EIMD-induced hamstring stiffening is not uniform across muscles, but is instead mediated by a complex interplay among neuromuscular activation strategies, architectural susceptibility to strain, and relative mechanical load distribution.

Based on the present findings, post-exercise cryotherapy protocols warrant comprehensive reevaluation. Although the present study applied a 20-min cooling session at each intervention time point, recent findings suggest that the optimal timing and frequency of application remain uncertain. For example, local cooling within the first 6 h post-injury may help attenuate pain, whereas application beyond 12 h could interfere with tissue healing (Racinais et al., 2024), and animal models have even shown prolonged pain behaviors following acute cryotherapy (Lima et al., 2026). However, because these previous studies primarily focused on pain and tissue inflammation, the optimal cooling protocol may vary depending on the outcome of interest, such as objective neuromuscular function or subjective recovery symptoms. Furthermore, as discussed above regarding the limitations of extrapolating findings from animal models of muscle damage to humans, small animals also differ substantially from humans in muscle volume, skin thickness, and subcutaneous fat, indicating that equivalent therapeutic doses cannot be directly inferred. Therefore, future human studies should systematically investigate different cooling durations, frequencies, and application times to determine whether optimized protocols can enhance recovery after EIMD.

Although certain methodological limitations, such as the relatively small sample size, have been noted above, several additional limitations of the present study warrant consideration. First, voluntary activation during MVIC was not quantified because of technical difficulties associated with applying electrical stimulation to the knee flexors. Consequently, the absence of twitch interpolation precludes confirmation of a true maximal voluntary effort and represents a potential source of bias in the interpretation of MVIC torque. Second, we did not assess biochemical markers, such as serum creatine kinase activity or interleukin-6 levels, which limited the mechanistic interpretation of the findings. Third, only a single intervention duration and delivery schedule were evaluated, and therefore, the effects of alternative cooling regimens remain unknown. Fourth, although group allocations were concealed from participants until the completion of the POST measurements, participant blinding during the subsequent intervention period and the use of sham controls were not feasible because of the distinct physical and thermal characteristics of the interventions. Furthermore, operational constraints precluded blinding of the examiners and analysts, which may have introduced observer bias despite the use of standardized testing protocols. Fifth, tissue and skin temperatures were not continuously monitored during the intervention, leaving potential differences in heat transfer between the cooling materials unquantified. Finally, the generalizability of the findings is limited because only healthy young men were included.

In conclusion, under the present experimental conditions, CDH cryotherapy administrated in 20-min sessions at designated recovery time points did not accelerate the recovery of muscle function or attenuate secondary markers following EIMD compared with conventional icing or no intervention. Consequently, these findings do not support the superiority of CDH cryotherapy over conventional icing or no intervention following eccentric exercise. To clarify whether CDH cryotherapy can provide practical therapeutic benefits for recovery from EIMD, future research should optimize application protocols in terms of cooling duration, frequency, and timing while evaluating more diverse populations beyond healthy young men.

## Data Availability

The raw data supporting the conclusions of this article will be made available by the authors, without undue reservation.

## References

[B1] Akagi R. Miyokawa Y. Shiozaki D. Yajima Y. Yamada K. Kano K. . (2024). Eight-week neuromuscular electrical stimulation training produces muscle strength gains and hypertrophy, and partial muscle quality improvement in the knee extensors. J. Sports Sci. 41, 2209–2228. doi: 10.1080/02640414.2024.2318540 38390833

[B2] Akagi R. Nomura Y. Kawashima C. Ito M. Oba K. Tsuchiya Y. . (2022). Trade-off between maximal power output and fatigue resistance of the knee extensors for older men. J. Aging Phys. Act. 30, 1003–1013. doi: 10.1123/japa.2021-0384 35453123

[B3] Akagi R. Sato S. Hirata N. Imaizumi N. Tanimoto H. Ando R. . (2020). Eight-week low-intensity squat training at slow speed simultaneously improves knee and hip flexion and extension strength. Front. Physiol. 11, 893. doi: 10.3389/fphys.2020.00893 32848848 PMC7396687

[B4] Algafly A. A. George K. P. (2007). The effect of cryotherapy on nerve conduction velocity, pain threshold and pain tolerance. Br. J. Sports Med. 41, 365–369. doi: 10.1136/bjsm.2006.031237 17224445 PMC2465313

[B5] Auley D. M. (2001). Ice therapy: How good is the evidence? Int. J. Sports Med. 22, 379–384. doi: 10.1055/s-2001-15656 11510876

[B6] Bourne M. N. Opar D. A. Williams M. D. Al Najjar A. Shield A. J. (2016). Muscle activation patterns in the Nordic hamstring exercise: Impact of prior strain injury. Scand. J. Med. Sci. Sports 26, 666–674. doi: 10.1111/sms.12494 26059634

[B7] Bourne M. N. Williams M. D. Opar D. A. Al Najjar A. Kerr G. K. Shield A. J. (2017). Impact of exercise selection on hamstring muscle activation. Br. J. Sports Med. 51, 1021–1028. doi: 10.1136/bjsports-2015-095739 27467123

[B8] Brusco C. M. Radaelli R. Neske R. Peñailillo L. E. Pinto R. S. (2022). Rate of torque development as an indirect marker of muscle damage in the knee flexors. Sport Sci. Health 18, 75–83. doi: 10.1007/s11332-021-00776-130311153

[B9] Chalchat E. Gaston A. F. Charlot K. Peñailillo L. Valdés O. Tardo-Dino P. E. . (2022). Appropriateness of indirect markers of muscle damage following lower limbs eccentric-biased exercises: A systematic review with meta-analysis. PloS One 17, e0271233. doi: 10.1371/journal.pone.0271233 35834532 PMC9282447

[B10] Chen T. C. Chen H. L. Tseng W. C. Chou T. Y. Tu J. H. Parcell A. C. . (2023). Contralateral versus ipsilateral protective effect against muscle damage of the elbow flexors and knee extensors induced by maximal eccentric exercise. Scand. J. Med. Sci. Sports 33, 2548–2560. doi: 10.1111/sms.14482 37642310

[B11] Chen T. C. Tseng W. C. Kang H. Y. Chan C. W. Chen H. L. Nosaka K. (2025). Effects of eccentric exercise of the extensors on eccentric exercise-induced muscle damage of the flexors in limbs. Med. Sci. Sports Exerc. 57, 970–983. doi: 10.1249/MSS.0000000000003622 39648360 PMC12147748

[B12] Cheung K. Hume P. A. Maxwell L. (2003). Delayed onset muscle soreness: Treatment strategies and performance factors. Sports Med. 33, 145–164. doi: 10.2165/00007256-200333020-00005 12617692

[B13] Clarkson P. M. Nosaka K. Braun B. (1992). Muscle function after exercise-induced muscle damage and rapid adaptation. Med. Sci. Sports Exerc. 24, 512–520. doi: 10.1249/00005768-199205000-00004 1569847

[B14] Cohen J. (1988). Statistical Power Analysis for the Behavioral Sciences. 2nd Ed (New Jersey: Lawrence Erlbaum Associates).

[B15] Doguet V. Nosaka K. Plautard M. Gross R. Guilhem G. Guével A. . (2016). Neuromuscular changes and damage after isoload versus isokinetic eccentric exercise. Med. Sci. Sports Exerc. 48, 2526–2535. doi: 10.1249/MSS.0000000000001042 27434079

[B16] Dourado M. A. A. Vieira D. C. L. Boullosa D. Bottaro M. (2023). Different time course recovery of muscle edema within the quadriceps femoris and functional performance after single- vs multi-joint exercises. Biol. Sport. 40, 767–774. doi: 10.5114/biolsport.2023.119984 37398959 PMC10286608

[B17] Ema R. Nosaka K. Kawashima R. Kanda A. Ikeda K. Akagi R. (2021). Muscle length influence on rectus femoris damage and protective effect in knee extensor eccentric exercise. Scand. J. Med. Sci. Sports 31, 597–609. doi: 10.1111/sms.13890 33249658 PMC7986188

[B18] Evangelidis P. E. Shan X. Otsuka S. Yang C. Yamagishi T. Kawakami Y. (2021). Hamstrings load bearing in different contraction types and intensities: A shear-wave and B-mode ultrasonographic study. PloS One 16, e0251939. doi: 10.1371/journal.pone.0251939 34010359 PMC8133428

[B19] Garrett W. E. Jr. (1996). Muscle strain injuries. Am. J. Sports Med. 24, S2–S8. doi: 10.1177/036354659602406S02 8947416

[B20] Goreau V. Pigne R. Bernier N. Nordez A. Hug F. Lacourpaille L. (2022). Hamstring muscle activation strategies during eccentric contractions are related to the distribution of muscle damage. Scand. J. Med. Sci. Sports 32, 1335–1345. doi: 10.1111/sms.14191 35611628 PMC9541962

[B21] Gronwald T. Klein C. Hoenig T. Pietzonka M. Bloch H. Edouard P. . (2022). Hamstring injury patterns in professional male football (soccer): A systematic video analysis of 52 cases. Br. J. Sports Med. 56, 165–171. doi: 10.1136/bjsports-2021-104769 34876406

[B22] Higashihara A. Ono T. Kubota J. Fukubayashi T. (2010). Differences in the electromyographic activity of the hamstring muscles during maximal eccentric knee flexion. Eur. J. Appl. Physiol. 108, 355–362. doi: 10.1007/s00421-009-1242-z 19816706

[B23] Hirata K. Shiozaki D. Yamada K. Miyokawa Y. Yajima Y. Akagi R. (2024). Cryotherapy with carbon dioxide hydrate enhances immediate recovery of muscle function from neuromuscular fatigue. J. Sports Sci. 42, 2103–2114. doi: 10.1080/02640414.2024.2423135 39533652

[B24] Hirata K. Tanimoto H. Sato S. Hirata N. Imaizumi N. Sugihara Y. . (2020a). Carbon dioxide hydrate as a recovery tool after fatigue of the plantar flexors. J. Biomech. 108, 109900. doi: 10.1016/j.jbiomech.2020.109900 32636011

[B25] Hirata K. Yamadera R. Akagi R. (2020b). Can static stretching reduce stiffness of the triceps surae in older men? Med. Sci. Sports Exerc. 52, 673–679. doi: 10.1249/MSS.0000000000002186 31652247 PMC7034366

[B26] Hody S. Rogister B. Leprince P. Laglaine T. Croisier J. L. (2013). The susceptibility of the knee extensors to eccentric exercise-induced muscle damage is not affected by leg dominance but by exercise order. Clin. Physiol. Funct. Imaging 33, 373–380. doi: 10.1111/cpf.12040 23701247

[B27] Horner D. Jeffries O. Brownstein C. G. (2024). Eccentric exercise–induced muscle damage reduces gross efficiency. Med. Sci. Sports Exerc. 56, 1816–1829. doi: 10.1249/MSS.0000000000003467 38768026

[B28] Kawashima M. Kawanishi N. Tominaga T. Suzuki K. Miyazaki A. Nagata I. . (2021). Icing after eccentric contraction-induced muscle damage perturbs the disappearance of necrotic muscle fibers and phenotypic dynamics of macrophages in mice. J. Appl. Physiol. (1985). 130, 1410–1420. doi: 10.1152/japplphysiol.01069.2020 33764172

[B29] Kawashima M. Nagata I. Terada E. Tamari A. Kurauchi M. Sakuraya T. . (2024). Frequent icing stimulates skeletal muscle regeneration following injury with necrosis in a small fraction of myofibers in rats. J. Histochem. Cytochem. 72, 569–584. doi: 10.1369/00221554241274882 39240248 PMC11456165

[B30] Klich S. Krymski I. Michalik K. Kawczyński A. (2018). Effect of short-term cold-water immersion on muscle pain sensitivity in elite track cyclists. Phys. Ther. Sport. 32, 42–47. doi: 10.1016/j.ptsp.2018.04.022 29738892

[B31] Knight K. L. Brucker J. B. Stoneman P. D. Rubley M. D. (2000). Muscle injury management with cryotherapy. Athl. Ther. Today 5, 26–30. doi: 10.1123/att.5.4.26

[B32] Kwiecien S. (2023). Is it the end of the ice age? Int. J. Sports Phys. Ther. 18, 547–550. doi: 10.26603/001c.74273 37425111 PMC10324284

[B33] Lacourpaille L. Nordez A. Hug F. Couturier A. Dibie C. Guilhem G. (2014). Time-course effect of exercise-induced muscle damage on localized muscle mechanical properties assessed using elastography. Acta Physiol. (Oxf). 211, 135–146. doi: 10.1111/apha.12272 24602146

[B34] Lima L. V. Pittman C. Laor B. Fouda I. Karaky M. Di Maria M. . (2026). Cryotherapy and duration of inflammatory pain in mice. Anesthesiology 145, 147–159. doi: 10.1097/aln.0000000000006066 41894397

[B35] Mawhinney C. Jones H. Joo C. H. Low D. A. Green D. J. Gregson W. (2013). Influence of cold-water immersion on limb and cutaneous blood flow after exercise. Med. Sci. Sports Exerc. 45, 2277–2285. doi: 10.1249/MSS.0b013e31829d8e2e 24240118

[B36] Nordez A. Hug F. (2010). Muscle shear elastic modulus measured using supersonic shear imaging is highly related to muscle activity level. J. Appl. Physiol. (1985). 108, 1389–1394. doi: 10.1152/japplphysiol.01323.2009 20167669

[B37] Ono T. Okuwaki T. Fukubayashi T. (2010). Differences in activation patterns of knee flexor muscles during concentric and eccentric exercises. Res. Sports Med. 18, 188–198. doi: 10.1080/15438627.2010.490185 20623435

[B38] Opar D. A. Williams M. D. Shield A. J. (2012). Hamstring strain injuries: Factors that lead to injury and re-injury. Sports Med. 42, 209–226. doi: 10.2165/11594800-000000000-00000 22239734

[B39] Peake J. M. Neubauer O. Della Gatta P. A. Nosaka K. (2017). Muscle damage and inflammation during recovery from exercise. J. Appl. Physiol. (1985). 122, 559–570. doi: 10.1152/japplphysiol.00971.2016 28035017

[B40] Racinais S. Dablainville V. Rousse Y. Ihsan M. Grant M. E. Schobersberger W. . (2024). Cryotherapy for treating soft tissue injuries in sport medicine: A critical review. Br. J. Sports Med. 58, 1215–1223. doi: 10.1136/bjsports-2024-108304 39237265

[B41] Sakai Y. Miwa M. Oe K. Ueha T. Koh A. Niikura T. . (2011). A novel system for transcutaneous application of carbon dioxide causing an “artificial Bohr effect” in the human body. PloS One 6, e24137. doi: 10.1371/journal.pone.0024137 21931656 PMC3169585

[B42] Shoji M. Ema R. Nosaka K. Kanda A. Hirata K. Akagi R. (2021). Muscle damage indicated by maximal voluntary contraction strength changes from immediately to 1 day after eccentric exercise of the knee extensors. Front. Physiol. 12, 775157. doi: 10.3389/fphys.2021.775157 34867482 PMC8635727

[B43] Smith D. H. Wilder J. W. Seshadri K. (2002). Thermodynamics of carbon dioxide hydrate formation in media with broad pore-size distributions. Environ. Sci. Technol. 36, 5192–5198. doi: 10.1021/es015862u 12523437

[B44] Takagi R. Fujita N. Arakawa T. Kawada S. Ishii N. Miki A. (2011). Influence of icing on muscle regeneration after crush injury to skeletal muscles in rats. J. Appl. Physiol. (1985). 110, 382–388. doi: 10.1152/japplphysiol.01187.2010 21164157

[B45] Vieira Ramos G. Pinheiro C. M. Messa S. P. Delfino G. B. Marqueti R. C. Salvini T. F. . (2016). Cryotherapy reduces inflammatory response without altering muscle regeneration process and extracellular matrix remodeling of rat muscle. Sci. Rep. 6, 18525. doi: 10.1038/srep18525 26725948 PMC4698758

[B46] Voglar M. Vatovec R. Kozinc Ž. Šarabon N. (2022). The effects of eccentric exercise on passive hamstring muscle stiffness: Comparison of shear-wave elastography and passive knee torque outcomes. Eur. J. Transl. Myol. 32, 10567. doi: 10.4081/ejtm.2022.10567 35666465 PMC9295161

[B47] Ward S. R. Lieber R. L. (2005). Density and hydration of fresh and fixed human skeletal muscle. J. Biomech. 38, 2317–2320. doi: 10.1016/j.jbiomech.2004.10.001 16154420

[B48] Wolska B. Domagała Ł. Kisilewicz A. Hassanlouei H. Makar P. Kawczyński A. . (2023). Multiple cryosauna sessions for post-exercise recovery of delayed onset muscle soreness (DOMS): a randomized control trial. Front. Physiol. 14, 1253140. doi: 10.3389/fphys.2023.1253140 37772056 PMC10523143

