# Supplementary material for: Effects of carbon dioxide hydrate cryotherapy on knee flexor muscle function recovery trajectory after muscle damage: a randomized controlled trial
Source: Front Physiol. 2026 Sep 22;17:1924770. doi: 10.3389/fphys.2026.1924770 (PMC13638424; doi:10.3389/fphys.2026.1924770)
Supplement: Supplementary file 1 [file Table1.docx]

**Supplementary Table S1.** Baseline-adjusted between-group differences for changes in knee flexion torque during maximal voluntary isometric contraction from baseline across post-exercise time points (n = 8 per group).

| Time | Contrast | Difference (Nm) | 95% CI (Nm) |
| --- | --- | --- | --- |
|  |  |  |  |
| POST | CDH vs ICE | −9.4 | −25.0, 6.1 |
|  | CDH vs CON | −12.3 | −28.4, 3.9 |
|  | ICE vs CON | −2.8 | −18.7, 13.1 |
|  |  |  |  |
| 4.5H | CDH vs ICE | −3.2 | −18.7, 12.4 |
|  | CDH vs CON | −4.1 | −20.2, 12.1 |
|  | ICE vs CON | −0.9 | −16.8, 14.9 |
|  |  |  |  |
| 24H | CDH vs ICE | 6.4 | −9.1, 22.0 |
|  | CDH vs CON | 2.5 | −13.6, 18.7 |
|  | ICE vs CON | −3.9 | −19.8, 12.0 |
|  |  |  |  |
| 48H | CDH vs ICE | 2.0 | −13.6, 17.5 |
|  | CDH vs CON | 6.5 | −9.6, 22.6 |
|  | ICE vs CON | 4.5 | −11.3, 20.4 |
|  |  |  |  |
| 72H | CDH vs ICE | 0.2 | −15.3, 15.8 |
|  | CDH vs CON | 8.6 | −7.6, 24.7 |
|  | ICE vs CON | 8.3 | −7.5, 24.2 |
|  |  |  |  |
| 168H | CDH vs ICE | 4.6 | −11.0, 20.1 |
|  | CDH vs CON | 3.6 | −12.6, 19.7 |
|  | ICE vs CON | −1.0 | −16.9, 14.9 |

CDH, carbon dioxide hydrate cryotherapy; ICE, conventional icing; CON, non-intervention control; CI, confidence interval; POST, immediately post-exercise; 4.5H, 4.5 h post-exercise; 24H, 24 h post-exercise; 48H, 48 h post-exercise; 72H, 72 h post-exercise; 168H, 168 h post-exercise.
