# Supplementary material for: Effects of carbon dioxide hydrate cryotherapy on knee flexor muscle function recovery trajectory after muscle damage: a randomized controlled trial
Source: Front Physiol. 2026 Sep 22;17:1924770. doi: 10.3389/fphys.2026.1924770 (PMC13638424; doi:10.3389/fphys.2026.1924770)
Supplement: Supplementary file 2 [file Table2.docx]

**Supplementary Table S2.** Baseline-adjusted between-group differences for changes in thigh circumference from baseline across post-exercise time points (n = 8 per group).

| Time | Contrast | Difference (cm) | 95% CI (cm) |
| --- | --- | --- | --- |
|  |  |  |  |
| 4.5H | CDH vs ICE | 0.1 | −1.1, 1.2 |
|  | CDH vs CON | −0.3 | −1.4, 0.9 |
|  | ICE vs CON | −0.3 | −1.5, 0.8 |
|  |  |  |  |
| 24H | CDH vs ICE | 0.2 | −1.0, 1.3 |
|  | CDH vs CON | 0.8 | −0.3, 1.9 |
|  | ICE vs CON | 0.6 | −0.5, 1.8 |
|  |  |  |  |
| 48H | CDH vs ICE | 0.4 | −0.7, 1.6 |
|  | CDH vs CON | 0.2 | −0.9, 1.4 |
|  | ICE vs CON | −0.2 | −1.3, 0.9 |
|  |  |  |  |
| 72H | CDH vs ICE | 0.3 | −0.8, 1.4 |
|  | CDH vs CON | 0.4 | −0.7, 1.5 |
|  | ICE vs CON | 0.1 | −1.0, 1.2 |
|  |  |  |  |
| 168H | CDH vs ICE | 0.3 | −0.9, 1.4 |
|  | CDH vs CON | −0.6 | −1.7, 0.5 |
|  | ICE vs CON | −0.9 | −2.0, 0.3 |

CDH, carbon dioxide hydrate cryotherapy; ICE, conventional icing; CON, non-intervention control; CI, confidence interval; 4.5H, 4.5 h post-exercise; 24H, 24 h post-exercise; 48H, 48 h post-exercise; 72H, 72 h post-exercise; 168H, 168 h post-exercise.
