# Supplementary material for: Effects of carbon dioxide hydrate cryotherapy on knee flexor muscle function recovery trajectory after muscle damage: a randomized controlled trial
Source: Front Physiol. 2026 Sep 22;17:1924770. doi: 10.3389/fphys.2026.1924770 (PMC13638424; doi:10.3389/fphys.2026.1924770)
Supplement: Supplementary file 3 [file Table3.docx]

**Supplementary Table S3.** Baseline-adjusted between-group differences for changes in the muscle shear moduli of in the long head of the biceps femoris (BF), semitendinosus (ST), and semimembranosus (SM) from baseline across post-exercise time points (n = 8 per group).

| Muscle | Time | Contrast | Difference (kPa) | 95% CI (kPa) |
| --- | --- | --- | --- | --- |
|  |  |  |  |  |
| BF | 4.5H | CDH vs ICE | 0.30 | −0.46, 1.05 |
|  |  | CDH vs CON | −0.12 | −0.88, 0.64 |
|  |  | ICE vs CON | −0.42 | −1.17, 0.34 |
|  |  |  |  |  |
|  | 24H | CDH vs ICE | 0.18 | −0.57, 0.94 |
|  |  | CDH vs CON | 0.24 | −0.52, 0.99 |
|  |  | ICE vs CON | 0.05 | −0.71, 0.81 |
|  |  |  |  |  |
|  | 48H | CDH vs ICE | 0.17 | −0.59, 0.92 |
|  |  | CDH vs CON | −0.19 | −0.94, 0.57 |
|  |  | ICE vs CON | −0.35 | −1.11, 0.41 |
|  |  |  |  |  |
|  | 72H | CDH vs ICE | 0.22 | −0.54, 0.98 |
|  |  | CDH vs CON | −0.20 | −0.96, 0.55 |
|  |  | ICE vs CON | −0.42 | −1.18, 0.34 |
|  |  |  |  |  |
|  | 168H | CDH vs ICE | −0.04 | −0.79, 0.72 |
|  |  | CDH vs CON | 0.41 | −0.35, 1.17 |
|  |  | ICE vs CON | 0.45 | −0.31, 1.21 |
|  |  |  |  |  |
| ST | 4.5H | CDH vs ICE | 0.39 | −0.30, 1.09 |
|  |  | CDH vs CON | −0.28 | −0.93, 0.38 |
|  |  | ICE vs CON | −0.67 | −1.37, 0.04 |
|  |  |  |  |  |
|  | 24H | CDH vs ICE | −0.09 | −0.78, 0.60 |
|  |  | CDH vs CON | −0.20 | −0.86, 0.46 |
|  |  | ICE vs CON | −0.11 | −0.81, 0.60 |
|  |  |  |  |  |
|  | 48H | CDH vs ICE | 0.05 | −0.65, 0.74 |
|  |  | CDH vs CON | −0.08 | −0.74, 0.57 |
|  |  | ICE vs CON | −0.13 | −0.83, 0.57 |
|  |  |  |  |  |
|  | 72H | CDH vs ICE | 0.23 | −0.47, 0.92 |
|  |  | CDH vs CON | 0.23 | −0.42, 0.89 |
|  |  | ICE vs CON | 0.01 | −0.70, 0.71 |
|  |  |  |  |  |
|  | 168H | CDH vs ICE | 0.31 | −0.38, 1.00 |
|  |  | CDH vs CON | 0.11 | −0.54, 0.77 |
|  |  | ICE vs CON | −0.20 | −0.90, 0.51 |
|  |  |  |  |  |
| SM | 4.5H | CDH vs ICE | −0.16 | −0.93, 0.62 |
|  |  | CDH vs CON | −0.17 | −0.95, 0.61 |
|  |  | ICE vs CON | 0.32 | −0.48, 1.13 |
|  |  |  |  |  |
|  | 24H | CDH vs ICE | 0.35 | −0.42, 1.13 |
|  |  | CDH vs CON | 0.02 | −0.76, 0.80 |
|  |  | ICE vs CON | −0.34 | −1.14, 0.47 |
|  |  |  |  |  |
|  | 48H | CDH vs ICE | −0.17 | −0.95, 0.60 |
|  |  | CDH vs CON | −0.21 | −0.99, 0.57 |
|  |  | ICE vs CON | 0.38 | −0.42, 1.19 |
|  |  |  |  |  |
|  | 72H | CDH vs ICE | −0.19 | −0.96, 0.59 |
|  |  | CDH vs CON | −0.35 | −1.13, 0.43 |
|  |  | ICE vs CON | 0.53 | −0.27, 1.34 |
|  |  |  |  |  |
|  | 168H | CDH vs ICE | −0.12 | −0.90, 0.65 |
|  |  | CDH vs CON | −0.65 | −1.43, 0.13 |
|  |  | ICE vs CON | 0.77 | −0.03, 1.57 |

CDH, carbon dioxide hydrate cryotherapy; ICE, conventional icing; CON, non-intervention control; CI, confidence interval; 4.5H, 4.5 h post-exercise; 24H, 24 h post-exercise; 48H, 48 h post-exercise; 72H, 72 h post-exercise; 168H, 168 h post-exercise.
