# Supplementary material for: Effects of carbon dioxide hydrate cryotherapy on knee flexor muscle function recovery trajectory after muscle damage: a randomized controlled trial
Source: Front Physiol. 2026 Sep 22;17:1924770. doi: 10.3389/fphys.2026.1924770 (PMC13638424; doi:10.3389/fphys.2026.1924770)
Supplement: Supplementary file 4 [file Table4.docx]

**Supplementary Table S4.** Baseline-adjusted between-group differences for changes in pressure-induced muscle soreness assessed using a visual analog scale in the long head of the biceps femoris (BF), semitendinosus (ST), and semimembranosus (SM) from baseline across post-exercise time points (n = 8 per group).

| Muscle | Time | Contrast | Difference (cm) | 95% CI (cm) |
| --- | --- | --- | --- | --- |
|  |  |  |  |  |
| BF | 4.5H | CDH vs ICE | 0.2 | −1.3, 1.7 |
|  |  | CDH vs CON | 0.0 | −1.6, 1.5 |
|  |  | ICE vs CON | −0.2 | −1.8, 1.3 |
|  |  |  |  |  |
|  | 24H | CDH vs ICE | 0.4 | −1.1, 1.9 |
|  |  | CDH vs CON | −0.8 | −2.3, 0.8 |
|  |  | ICE vs CON | −1.2 | −2.7, 0.3 |
|  |  |  |  |  |
|  | 48H | CDH vs ICE | 0.1 | −1.4, 1.7 |
|  |  | CDH vs CON | −1.0 | −2.5, 0.5 |
|  |  | ICE vs CON | −1.1 | −2.7, 0.4 |
|  |  |  |  |  |
|  | 72H | CDH vs ICE | 0.5 | −1.0, 2.0 |
|  |  | CDH vs CON | −0.9 | −2.4, 0.6 |
|  |  | ICE vs CON | −1.4 | −2.9, 0.2 |
|  |  |  |  |  |
|  | 168H | CDH vs ICE | 1.0 | −0.6, 2.5 |
|  |  | CDH vs CON | 0.8 | −0.7, 2.3 |
|  |  | ICE vs CON | −0.2 | −1.7, 1.4 |
|  |  |  |  |  |
| ST | 4.5H | CDH vs ICE | 0.2 | −1.0, 1.4 |
|  |  | CDH vs CON | 0.1 | −1.1, 1.2 |
|  |  | ICE vs CON | −0.1 | −1.3, 1.1 |
|  |  |  |  |  |
|  | 24H | CDH vs ICE | −0.6 | −1.8, 0.6 |
|  |  | CDH vs CON | 0.0 | −1.1, 1.2 |
|  |  | ICE vs CON | 0.7 | −0.5, 1.9 |
|  |  |  |  |  |
|  | 48H | CDH vs ICE | 0.3 | −0.9, 1.5 |
|  |  | CDH vs CON | −0.4 | −1.5, 0.8 |
|  |  | ICE vs CON | −0.7 | −1.9, 0.5 |
|  |  |  |  |  |
|  | 72H | CDH vs ICE | −0.5 | −1.7, 0.7 |
|  |  | CDH vs CON | −0.1 | −1.3, 1.1 |
|  |  | ICE vs CON | 0.4 | −0.8, 1.6 |
|  |  |  |  |  |
|  | 168H | CDH vs ICE | 0.2 | −1.0, 1.4 |
|  |  | CDH vs CON | 0.6 | −0.6, 1.8 |
|  |  | ICE vs CON | 0.4 | −0.7, 1.6 |
|  |  |  |  |  |
| SM | 4.5H | CDH vs ICE | −0.2 | −1.6, 1.2 |
|  |  | CDH vs CON | −0.2 | −1.5, 1.1 |
|  |  | ICE vs CON | 0.0 | −1.4, 1.4 |
|  |  |  |  |  |
|  | 24H | CDH vs ICE | −0.4 | −1.7, 1.0 |
|  |  | CDH vs CON | 0.6 | −0.7, 1.9 |
|  |  | ICE vs CON | 0.9 | −0.5, 2.4 |
|  |  |  |  |  |
|  | 48H | CDH vs ICE | −0.2 | −1.5, 1.2 |
|  |  | CDH vs CON | 0.4 | −0.9, 1.7 |
|  |  | ICE vs CON | 0.6 | −0.8, 2.0 |
|  |  |  |  |  |
|  | 72H | CDH vs ICE | 0.0 | −1.3, 1.4 |
|  |  | CDH vs CON | 0.7 | −0.6, 2.0 |
|  |  | ICE vs CON | 0.7 | −0.7, 2.1 |
|  |  |  |  |  |
|  | 168H | CDH vs ICE | −0.1 | −1.4, 1.3 |
|  |  | CDH vs CON | 0.8 | −0.5, 2.1 |
|  |  | ICE vs CON | 0.8 | −0.6, 2.2 |

CDH, carbon dioxide hydrate cryotherapy; ICE, conventional icing; CON, non-intervention control; CI, confidence interval; 4.5H, 4.5 h post-exercise; 24H, 24 h post-exercise; 48H, 48 h post-exercise; 72H, 72 h post-exercise; 168H, 168 h post-exercise.
