# Supplementary material for: Effects of carbon dioxide hydrate cryotherapy on knee flexor muscle function recovery trajectory after muscle damage: a randomized controlled trial
Source: Front Physiol. 2026 Sep 22;17:1924770. doi: 10.3389/fphys.2026.1924770 (PMC13638424; doi:10.3389/fphys.2026.1924770)
Supplement: Supplementary file 5 [file Table5.docx]

**Supplementary Table S5.** Baseline-adjusted between-group differences for changes in hip flexion range of motion from baseline across post-exercise time points (n = 8 per group).

| Time | Contrast | Difference (°) | 95% CI (°) |
| --- | --- | --- | --- |
|  |  |  |  |
| 4.5H | CDH vs ICE | −7.3 | −16.6, 2.0 |
|  | CDH vs CON | −17.5 | −27.2, −7.8 |
|  | ICE vs CON | −10.2 | −19.4, −1.1 |
|  |  |  |  |
| 24H | CDH vs ICE | −0.7 | −10.0, 8.6 |
|  | CDH vs CON | −7.7 | −17.4, 2.0 |
|  | ICE vs CON | −7.0 | −16.2, 2.1 |
|  |  |  |  |
| 48H | CDH vs ICE | −10.5 | −19.8, −1.2 |
|  | CDH vs CON | −12.5 | −22.2, −2.8 |
|  | ICE vs CON | −2.0 | −11.2, 7.1 |
|  |  |  |  |
| 72H | CDH vs ICE | −7.6 | −16.9, 1.7 |
|  | CDH vs CON | −8.3 | −17.9, 1.4 |
|  | ICE vs CON | −0.7 | −9.8, 8.5 |
|  |  |  |  |
| 168H | CDH vs ICE | −0.1 | −9.4, 9.3 |
|  | CDH vs CON | −5.4 | −15.1, 4.3 |
|  | ICE vs CON | −5.4 | −14.5, 3.8 |

CDH, carbon dioxide hydrate cryotherapy; ICE, conventional icing; CON, non-intervention control; CI, confidence interval; 4.5H, 4.5 h post-exercise; 24H, 24 h post-exercise; 48H, 48 h post-exercise; 72H, 72 h post-exercise; 168H, 168 h post-exercise.
